# Case of Excision of the Upper Jaw

**Published:** 1869-01

**Authors:** 


### ARTICLE VII.

### *Case of Excision of the Upper Jaw.*

(Under the care of Dr. D. B. Reid.)

For the following notes we are indebted to Mr. John Douglas, clinical clerk :—

William B——, farm labourer, native of Chillwel, a tall thin young man, with pale complexion, grey eyes, and brown hair, was admitted July 15th, 1867, suffering from tumour of right upper jaw.

He states that, twelve months ago, he was thrown from a horse, and fell on the right side of his cheek and head. A week afterwards a small pimple formed in the centre of his cheek, which has gradually grown, and swelled to an enormous size.

There is a large tumour occupying the site of the right upper maxillary bone. A solid irregular mass appears to grow outwards and downwards from the bone, distending the cheek. It is hard and botryoidal. The soft palate is pushed downwards, as if covering a tumour in this direction. Both nares are perfectly closed; there is no passage, and the air cannot be sniffed up or expelled. He suffers no pain at all. There is no depression of the hard palate, and the skin is nowhere adhernt. The zygomatic arch appears more prominent than on the left side. The patient is rather weak, but feels very well. On introducing the finger into the mouth, above and behind the soft palate, a mass, tough, lobulated, and apparently non-adherent, is found, blocking up the posterior nares, depressing the soft palate, and projecting into the pharynx. Ordered spoon diet—one pint of beef-tea, half a pint of milk, two ounces of arrowroot, one ounce of sugar; mutton chop, and four ounces of brandy.

*Operation.*—On the 14th of August, the patient being in good condition, the operation was performed. The man was placed in an arm-chair, an assistant supporting the head. The lines of incision were completely frozen by ether spray. On dissecting the face up in the usual way, a large fibrous tumour was found under the cheek, and the superior maxillary bone was found expanded: and it was clear that the disease commenced in the antrum. Incisons were made through the zygoma and malar bone, and from the nose into the orbit, as well as from the nose into the mouth, by means of Hey's saw. The hard palate was so thin that, with a scalpel, the cut made by the saw was easily prolonged as far as necessary. A few taps with the hammer readily started the bone; but it could not easily be removed in consequence of the deep attachments of the tumour. The soft palate was then dissected from the bone and from the tumour. The tumour, however, had contracted deeper and firm adhesions to the body of the sphenoid and the to pterygoid process of the right side. This dissection was very tedious and difficult. The last band of the fibres, which went very near the carotid artery, was cut through by an ecraseur. The whole of the right superior maxillary, the malar, and the palate, and a small portion of pterygoid process were thus removed together with a large lobulated fibrous tumour, which implicated them all. Chloroform was tried, but abandoned. Two bloodvessels were tied, and the ends cut off short. The hæmorrhage was copious. The inner incision was brought together at once, but the outer not until four hours after.

*Dissection.*—The tumour was perfectly white, lobulated, very tough and fibrous, and the mass weighed ten ounces. It resembled a sweetbread, was lobulated and irregular. The antrum was enlarged, distended, and contained one lobe of the tumour, of the size and shape of a large walnut. It communicated with the mass outside the bone and in the cheek, and also with a large mass in the nasal cavity and back of the throat. The tumour extended not only into the back of the throat, but also had further attachments to the under surface of the body of the sphenoid bone. The tumor appeared to have originated in the antrum, and to have spread from there into the nasal cavities, which were completely occupied, back into the pharynx, and then outwards behind the pterygoid process to the cheek. On section, the tumour was beautifully white, exceedingly tough, and microscopically fibrous.

Aug. 27th.—The wound all but completely healed up, and the patient walking about the ward.—*Lancet.*

